# Mind wandering in text comprehension under dual-task conditions

**DOI:** 10.3389/fpsyg.2013.00682

**Published:** 2013-10-01

**Authors:** Peter Dixon, Henry Li

**Affiliations:** Department of Psychology, University of AlbertaEdmonton, AB, Canada

**Keywords:** mind wandering, text comprehension, dual-task methodology, letter detection, missing-letter effect

## Abstract

In two experiments, subjects responded to on-task probes while reading under dual-task conditions. The secondary task was to monitor the text for occurrences of the letter e. In Experiment 1, reading comprehension was assessed with a multiple-choice recognition test; in Experiment 2, subjects recalled the text. In both experiments, the secondary task replicated the well-known “missing-letter effect” in which detection of e's was less effective for function words and the word “the.” Letter detection was also more effective when subjects were on task, but this effect did not interact with the missing-letter effect. Comprehension was assessed in both the dual-task conditions and in control single-task conditions. In the single-task conditions, both recognition (Experiment 1) and recall (Experiment 2) was better when subjects were on task, replicating previous research on mind wandering. Surprisingly, though, comprehension under dual-task conditions only showed an effect of being on task when measured with recall; there was no effect on recognition performance. Our interpretation of this pattern of results is that subjects generate responses to on-task probes on the basis of a retrospective assessment of the contents of working memory. Further, we argue that under dual-task conditions, the contents of working memory is not closely related to the reading processes required for accurate recognition performance. These conclusions have implications for models of text comprehension and for the interpretation of on-task probe responses.

Mind wandering can be characterized as the allocation of resources to mental processes unrelated to the current task. In the present research, we explored this conception of mind wandering by using a dual-task paradigm, a common technique for indexing the allocation of resources. The primary task was reading comprehension, and we measured the effectiveness of comprehension with either a multiple-choice test (Experiment 1) or recall (Experiment 2). The secondary task was to monitor the words being read for the occurrence of the letter e. Although this task requires the recognition of individual words, it depends on orthographic processing rather than text comprehension. Thus, letter detection may interact with comprehension, which in turn may interact with whether subjects are on or off task. The surprising result was that there was no relationship between on-task rating and comprehension when measured with a recognition test, although this relationship was found under either single-task conditions or when comprehension was measured by recall. This result has implications for the nature and measurement of mind wandering.

The plan of this article is as follows. First, we describe in more detail our analysis of mind wandering in terms of resource allocation. Second, this analysis is applied to the mental processes involved in reading comprehension, and previous results are described from this perspective. Third, we describe the letter-detection task, the missing-letter phenomenon, and how the task likely relates to reading for comprehension. Fourth, we present the results of the two experiments comparing the effects of mind wandering on comprehension under single- to dual-task conditions. Experiment 1 used a multiple-choice recognition task to assess memory for the text while Experiment 2 used a recall task. In both experiments, we used periodic mental-state probes to assess whether subjects were on or off task. In the Discussion section, we argue that the pattern of results requires a new analysis of measures of mind wandering.

## Mind wandering as resource allocation

While mind wandering, one can superficially conform to the demands of a task while devoting potentially large amounts of mental resources to other, unrelated processing. Thus, if one divides mental processing into that which is task relevant and that which is task irrelevant, mind wandering consists of devoting a significant portion of mental resources to task-irrelevant processing at the expense of the target task (Smallwood et al., 2011). On the assumption that one wants to allocate resources to the current task, allocating resources elsewhere can be regarded as a failure of executive control (cf. McVay and Kane, 2009). However, mind wandering might also occur because task-irrelevant goals are more important, at least momentarily (e.g., Smallwood and Schooler, 2006). Related to this analysis, mind wandering has been characterized as directing resources to internal thoughts rather the external stimuli needed to perform a task (Smallwood, 2013). However, in the present research, we were concerned with minding wandering during the task of reading comprehension, and in this case the distinction between directing resources to external vs. internal information seems incomplete. In reading, as in many other complex tasks, resources must be directed to the contents of internal processing as well as external stimuli. Based on this premise, Dixon and Bortolussi (2013) analyzed mind wandering in text comprehension as part of a more general issue of resource allocation. Previous research suggests that mind wandering in reading has a surprisingly high prevalence (Schooler et al., 2004), making an understanding of this aspect of mental processing an important problem in understanding the use of mental resources in reading.

## Component processes in reading

We argue that for the task of reading comprehension, it is simplistic to think that resources are either allocated to the task of reading or not. Rather, reading consists of a variety of different component processes, and resources can be allocated differentially among them (cf. Smallwood, 2011; Schad et al., 2012). As a working heuristic, we distinguish among lexical processes, meaning processes, and situation model processes. Lexical processes are those that use the orthographic information in the text to identify the word and provide initial access to aspects of the word meaning. The importance of resource allocation to the lexical level is highlighted by the classic work of LaBerge and Samuels (1974). They argued that the development of automatic word recognition skill among developing readers allowed resources to be devoted instead to higher-level comprehension processes. For our purposes, we distinguish two general classes of processes in reading beyond word recognition. Meaning processes are responsible for constructing a propositional representation of the word meanings and semantic content, the so-called text base (Kintsch and Van Dijk, 1978). In this context, the text base refers to the relationships among concepts and events that are depicted by sentences and referring expressions in the text. Meaning processes correspond to the construction processes in the Construction-Integration Model of reading (Kintsch and Welsch, 1991). In contrast, situation model processes construct a representation of that to which the text refers. Zwaan et al. (1995) suggested that the situation model is “a coherent representation of the described world” (p. 292). It would include, for example, information about the protagonist's goals and how they are related to other aspects of the narrative world, the spatial and temporal relationships among the entities in the story, and so on. Generally, construction of a situation model requires inferences based on world knowledge and personal experience that go beyond the information provided explicitly by the text.

The three levels of processing—lexical, meaning, and situation model—interact and build on one another. For example, a representation of the word meanings is necessary for the construction of the text base, and the text base is necessary for construction of the situation model. However, the component processes have different relationships to measures of subsequent memory. For example, lexical processing may be critical in verbatim recall since one must distinguish precisely which word was read. Meaning processing underlies memory for content. For example, a representation of the meaning would be involved if one had to distinguish which of two similar events occurred in a story. Situation-model processing allows readers to make inferences in the world described by the text. Because the situation model provides an organizational memory structure for the content of the story, it is essential for recall (Ericsson and Kintsch, 1995). Schmalhofer and Glavanov (1986) demonstrated differential memory for these representations, with verbatim memory decaying quickly, propositional memory more slowly, and situation model memory lasting the longest (see also Singer and Kintsch, 2001).

There is evidence that each of these three components of reading could suffer during mind wandering. According to several accounts of reading comprehension, lexical processes are assumed to be largely automatic among skilled readers (e.g., Rayner and Pollatsek, 1989; Brown et al., 2002; cf. Perfetti, 2007; but see, e.g., Besner et al., 1997; Risko et al., 2005). Thus, one might expect that diverting resources to off-task processing while mind wandering to have relatively little impact on lexical processing. Indeed, lexical errors introduced into a text are detected with relatively high frequency, despite a propensity of readers to mind wander (Schad et al., 2012). On the other hand, word frequency effects are smaller during mind wandering (Reichle et al., 2010; Franklin et al., 2011; Schad et al., 2012), which might mean that lexical processes are less complete during mind wandering. Mind wandering may also produce decrements in either meaning processing or situation model processing. For example, Reichle et al. (2010) found that there were smaller effects of several lexical and sentential variables on eye fixations during reading when mind wandering, suggesting that processes that integrated word meanings into a propositional representation were operating less effectively. Similarly, Schad et al. (2012), using an objective index of mind wandering, found smaller sentence wrap-up effects (Just and Carpenter, 1980) when mind wandering, suggesting that processes that constructed sentence meaning were curtailed. If meaning representations were trapped in a subsequent comprehension test, decrements due to mind wandering might be found. Smallwood et al. (2008) argued that mind wandering had its main effect on the construction of the situation model. When adequate resources are not devoted to the construction of this representation, readers may be less able to draw inferences about the events in the world described by the text. In general, though, because lexical, meaning, and situation-model processes must interact extensively, it may be difficult to ascribe effects unambiguously to any one level.

## Letter detection

In the letter-detection task, subjects are asked to read for comprehension while at the same time identifying words with a particular letter. Although not always discussed as such, this is effectively a dual-task paradigm in which the comprehension must proceed at the same time as a novel orthographic task. A great deal of research using this task has focused on a result originally reported by Corcoran (1966) that has come to be known as the “missing-letter effect.” This result is that subjects disproportionately fail to detect letters in high-frequency function words. An influential explanation of the missing-letter effect is the Guidance-Organization, or GO, model (Greenberg et al., 2004). The GO model attributes the effect to a variety of factors, including unitization, in which high-frequency words are recognized before their constituent letters; parafoveal processing, in which words are recognized in the parafovea where there is inadequate acuity to identify individual letters; and contextual constraint, in which predictable function words need not be processed.

Letter detection and comprehension are related in that both require identification of the words of the text. However, letter detection requires attention to the details of the orthography and additional decision processes that would not otherwise be invoked (e.g., Saint-Aubin et al., 2003). Similarly, comprehension requires further processing of the sentence and discourse that is not required in making letter-detection responses. Although previous researchers have argued that mind wandering in reading is associated with less complete processing of the situation model, it is unclear how mind wandering should affect letter detection. One intuition is that the missing-letter effect occurs because readers fail to devote adequate resources to processing the orthographic level. If this were true, mind wandering should exacerbate the effect because readers would be even less likely to devote resources to letter detection. In other words, one might conjecture that detecting a letter in a high-frequency function word requires additional, non-automatic processing, and that such resources would be unavailable while mind wandering.

## Experiment 1

In the present research, we used a version of the on-task probe technique developed in the context of reading by Schooler et al. (2004). In this approach, subjects are occasionally interrupted while reading and asked whether or not they are on-task. Our implementation of this paradigm is similar to that described by Dixon and Bortolussi (2013) in which subjects used a computer mouse to indicate a point on a continuous scale to describe the extent to which they are on or off task. Thus, our measure of mind wandering ranges continuously from completely off task to completely on task.

In order to combine reading comprehension with letter detection, texts were read word by word on a computer screen. Subjects advanced from one word to the next by pressing the space bar. This word-by-word paradigm has been used in a variety of research on reading (e.g., Aaronson and Scarborough, 1976). In most situations, subjects readily acquire proficiency in controlling the presentation. Many variables that affect eye fixations in unconstrained reading also affect presentation time in this and similar paradigms (e.g., Just et al., 1982). When subjects were to read and to detect the letter e concurrently in the present experiment, they pressed the e key on the keyboard (rather than the spacebar) if the word being read contained an e. The dual-task condition also afforded the opportunity to assess whether subjects were on task with respect to letter detection as well as comprehension. Thus, in this condition, when subjects were interrupted, they were asked to respond to two on-task probes, one concerning comprehension and one concerning letter detection.

The use of a dual-task condition in this experiment constrained how we could assess whether subjects were on task. In the probe question used by, for example, Smallwood et al. (2007) and Schooler et al. (2004), subjects were asked directly whether they were mind wandering. As described above, mind wandering can be construed as devoting mental resources to task-unrelated thoughts. Thus, a positive response to such probe questions necessarily entails that resources were not devoted to the target task. However, in the current dual-task condition, there are two tasks to which subjects may or may not be devoting resources, and there may not be a simple relationship between mind wandering and the resources devoted to either of those tasks. Thus, our approach was to phrase the probe questions in terms of the target tasks. In other words, were they devoting resources to comprehension? And, were they devoting resources to letter detection? While such probe questions do not mention mind wandering, if mind wandering involves devoting resources to something other than the target task, the probes should provide the same information as that obtained in previous investigations.

A critical issue concerns how to assess reading comprehension. In Experiment 1, we used a multiple-choice test that indexed memory for relatively superficial details of the text. This type of test makes fairly minimal demands on the situation model readers might construct; it depends instead more on what concepts were activated in the course of reading. If mind wandering primarily affects the construction of a situation model (e.g., Smallwood et al., 2008; Smallwood, 2011), one might expect little relationship between this measure of comprehension and the on-task probe response. On the other hand, if mind wandering deflects resources from a variety of reading components, a relationship might be found.

### Method

Subjects read an extended, difficult passage word by word. In the single-task condition, this was the only task; in the dual-task condition, they concurrently monitored for words containing the letter e. In both conditions, subjects were periodically probed to see whether they were on task.

#### Materials

Subjects read the initial, 2040-word section of the introduction (MacDonell, 1910) to *The Closet of Sir Kenelm Digby Knight Opened*, a seventeenth century cookbook. The text describes the life and background of Sir Kenelm Digby using relatively difficult vocabulary and sentence structure. The text was edited slightly to eliminate a few words that were likely to be confusing to our subjects. It had a Flesch reading index of 59.8. The text was divided into eight approximately equal sections. The section lengths averaged 255 words, with a range of 194–368, and the section breaks always coincided with paragraph breaks. An on-task probe was presented after each section. Sixteen multiple-choice comprehension questions were presented after completing the reading task. Each question was based on the gist of a sentence in the second half of one of the eight sections and was designed to be difficult to answer unless the corresponding sentence had been understood. There were two questions for each section. As in other investigations of mind wandering in reading, we assume that the mental state indexed by the response to the probe question also applied to the processing of at least some of the text preceding the probe. However, we cannot be certain how long that mental state preceded the probe. As a working approximation, we examined comprehension of material in the second half of the words read since the preceding probe. Examples of the questions are presented in Table 1.

**Table 1:** **Examples of comprehension questions in Experiment 1**.

What best describes Kenelm's mother? **a. Broken and darkened** b. Warm and caring c. Witty and cunning d. Melancholic
According to the narrator, what contributed to the young Kenelm's career? **a. His restless mind** b. His excellent memory for dates and places c. His conservative nature d. His interest in history
Who was one of young Kenelm's tutors? a. John Digby, Earl of Bristol b. Sir Francis Drake **c. Laud, Dean of Gloucester** d. The Archbishop of Canterbury

*Answer in bold*.

#### Procedure

In the single-task condition, subjects viewed the text one word at a time, and the next word was presented each time the spacebar was pressed. Subjects read silently. Reading time for each word was measured. At the end of each section, there was a 0.5 s delay, and then a comprehension on-task probe was presented instead of the next word in the passage. The probe consisted of the question, “Were you fully comprehending the story or were you thinking of something else?” Subjects were informed of the probe questions at the beginning of the session and were given the following instructions:

We are also interested in how well people can concentrate on a task like this over the course of the research session. We are asking you to read a fairly long passage, and it is perfectly normal for people to occasionally think about other things rather than the passage and the task at hand. To check this, periodically the computer will display a question asking whether or not you are currently concentrating on reading.

Subjects responded to the probe by clicking with the mouse somewhere on a 16-cm horizontal line presented under the question. The line was marked with five points, labeled from left to right, “Definitely thinking of something else,” “Thinking of something else to some extent,” “Not sure,” “Comprehending to some extent,” and “Definitely comprehending.” Some of the scale labels have alternative interpretations. For example, one might imagine selecting “comprehending to some extent” if one were attending fully to the text but having comprehension difficulties. Similarly, “not sure” might be symptomatic of being “zoned out” and not attending to the task at all. However, we believe that the framing of the probe question as a continuous range from “comprehending” to “thinking of something else,” as well as the instructions provided at the beginning of the session, made it clear that subjects were to estimate the extent to which they are devoting resources to reading. The dependent probe measure was the position of the mouse click along the line, measured in pixels from −225 to 225.

The procedure in the dual-task condition was similar. However, subjects were asked to press the e key on the keyboard to advance to the next word (rather than the spacebar) if the current word contained an e. On probe trials, a probe pertaining to the letter-detection task was presented after the comprehension on-task probe and before continuing with the passage. (The order of the two probe questions was always the same.) The letter-detection on-task probe question was, “Were you carefully watching for e's or were you thinking of something else?” Subjects responded with the mouse as before; the response scale was marked with the labels, “Definitely thinking of something else,” “Thinking of something else to some extent,” “Not sure,” “Watching for e's to some extent,” and “Definitely watching for e's.”

Immediately after completing the reading task, subjects were given the comprehension questions on a printed page. Subjects answered each item by circling the correct alternative. Subjects were informed at the beginning of the task of the nature of the comprehension questions (cf. Postman and Senders, 1946).

#### Subjects

Subjects were undergraduate volunteers who participated as part of a Psychology course requirement. There were nine subjects each in the single- and dual-task conditions. One further subject in dual-task condition did not complete the comprehension test, and data from an additional subject in the dual-task condition was not used because the subject failed to follow the instruction to monitor for e's. The treatment of subjects, including procedures for obtaining informed consent, was approved by the Arts, Science, and Law Research Ethics Board at the University of Alberta according to the provisions of the Canadian Tri-Council Policy Statement, “Ethical Conduct for Research Involving Humans.”

#### Analysis

We did not use null-hypothesis significance testing because of the logical, interpretational, and pragmatic problems with this practice (e.g., Cohen, 1994; Dixon, 2003; Wagenmakers, 2007). Instead, we fit models to the data that embodied different possible interpretations of the results and compared the models using likelihood ratios. The likelihood ratio indicates the likelihood of the data given the best fit of one model relative to the likelihood of the data given the other. Thus, very large (or very small) values of the likelihood ratio indicate that the fit of one model is substantially better than the other. Likelihood ratios are readily calculated from the summary statistics for a model fit. For example, output from model fits in the environment R (R Development Core Team, 2012) typically include the log likelihood of the data given the best fit of the model. Thus, the likelihood ratio comparing two models would be:

$$\lambda \text{ = }{\text{e}}^{l_{1}-l_{2}}$$

where *l*_1_ and *l*_2_ are the log likelihoods of the models under consideration. Following the suggestion of Glover and Dixon (2004), the likelihood ratios were adjusted for the differing numbers of parameters based on the Akaike Information Criterion (AIC; Akaike, 1973). The adjustment has the form:

$$\lambda_{\text{adj}}{\text{= e}}^{l_{1}-l_{2}}/{\text{e}}^{k_{1}-k_{2}}$$

where *k*_1_ and *k*_2_ are the number of parameters in the two models. Comparing models based on an adjusted likelihood ratio is tantamount to model selection based on *AIC* values. In particular, an adjusted likelihood ratio can be calculated directly from the *AIC* values commonly provided for model fits, as in:

$$\lambda_{\text{adj}}=\exp\left(\frac{AIC_{2}-AIC_{1}}{2}\right)$$

Burnham and Anderson (2002) refer to this statistic as an evidence ratio. Adjusted likelihood ratios have an intuitive interpretation: After compensating for differential model flexibility, how much more likely are the data given one interpretation than they are given the other? In this form, the likelihood ratio provides a description of the evidence in favor of one model relative to the other. Thus, it should be intuitively clear when the magnitude of the likelihood ratio provides strong support for a given model. However, in some prototypical hypothesis testing situations, an attained significance level of 0.05 corresponds to an adjusted likelihood ratio of ~3. We use the symbol λ_adj_ to indicate the adjusted likelihood ratio.

The models were fit using mixed-effects analysis using the package lme4 (Bates et al., 2013) in the R environment (R Development Core Team, 2012). In mixed-effects analysis, one must explicitly identify the random effects. In analyzing our data, we assumed that the overall level of the response varied with subjects and, where applicable, text section. Comprehension accuracy and letter detection performance were analyzed using generalized linear models using the binomial family and a logistic link function as suggested by Dixon (2008).

### Results and discussion

#### On-task probes

Responses to the probes were in pixels (corresponding to the position on the response scale that subjects clicked), ranging from −225 to +225. The mean response for the comprehension on-task probe was −75.8 (*SE* = 32.8 across subjects) in the dual-task condition and 9.0 (*SE* = 36.1 across subjects) in the single-task condition. We compared a linear mixed model of the probe responses that incorporated a difference between the single- and dual-task conditions to a null model in which both conditions were assumed to be the same. The comparison indicated that the former was only slightly better (λ_adj_ = 1.74). Thus, there was little evidence that the responses were higher in the single task condition. The standard deviation of responses within subjects was 111.6. The mean response for the letter-detection on-task probe was −19.1 (*SE* = 40.5), and the standard deviation within subjects was 114.1. In order to assess whether there was a relationship between the two on-task responses, a model was constructed in which comprehension on-task response was predicted as a function of letter-detection on-task response. There was a weak positive relationship, and the model was somewhat better than a null model (λ_adj_ = 3.05). Another way to index the relationship between the two responses is to examine the correlation. However, in order to remove the contribution of subjects to this correlation, we first subtracted the mean comprehension response and mean detection response for each subject from the responses. With this correction, the correlation between the letter-detection response and the comprehension response was *r* = 0.232.

These results provide some confidence that the manipulation of dual- and single-task conditions did not influence the on-task probe responses as an index of mind wandering. The overall level of on-task responses was comparable in the single- and dual-task conditions, as was the range of responses. Further, subjects seemed to be able to respond to both the comprehension and letter-detection on-task probes without any obvious interference. The relationship between the responses to the two on-task probes is likely determined by two opposing tendencies: When subjects devote resources to off-task processes, there would be fewer resources for both comprehension and letter detection, leading to a positive correlation. On the other hand, resources that are devoted to on-task processing would need to be split between letter detection and comprehension; in this case, the two tasks may compete for resources, leading to a negative correlation. On balance, the modest positive relationship that was obtained is not surprising.

#### Comprehension

As described above, accuracy on the comprehension questions was analyzed in terms of generalized linear mixed-effects models, an approach tantamount to logistic regression. In the case, the dependent variable and model parameters are expressed in terms of log odds correct (i.e., log *p*/(1 − *p*), where *p* is the proportion correct). Accuracy and the corresponding proportion correct is shown in Table 2. (In these and subsequent tables, the means and standard errors were calculated by combining the parameter estimates in a full model of the data that includes all effects and interactions. Proportion correct were then calculated by back transforming the logit values. The full model was used in order to provide a detailed and unbiased description of the results even though our preferred interpretation of the data was typically simpler.) To assess the evidence for a difference in performance between the single- and dual-task conditions, a model of accuracy as a function task condition was compared to a null model. There was no evidence that condition had an effect (λ_adj_ = 0.38). Previous research on the letter-detection task has similarly failed to demonstrate strong interference with comprehension (Oliver et al., 2005). However, as discussed below, reading is substantially slower in the dual-task condition, and it is possible that the increase in time is due to the additional work involved in performing two tasks at the same time.

**Table 2:** **Comprehension accuracy (and Standard Error) in Experiment**.

**Condition**	**Log odds correct**	**Proportion correct**
Single task	0.195 (0.191)	0.548 (0.047)
Dual task	0.300 (0.191)	0.574 (0.046)

**Figure 1:**
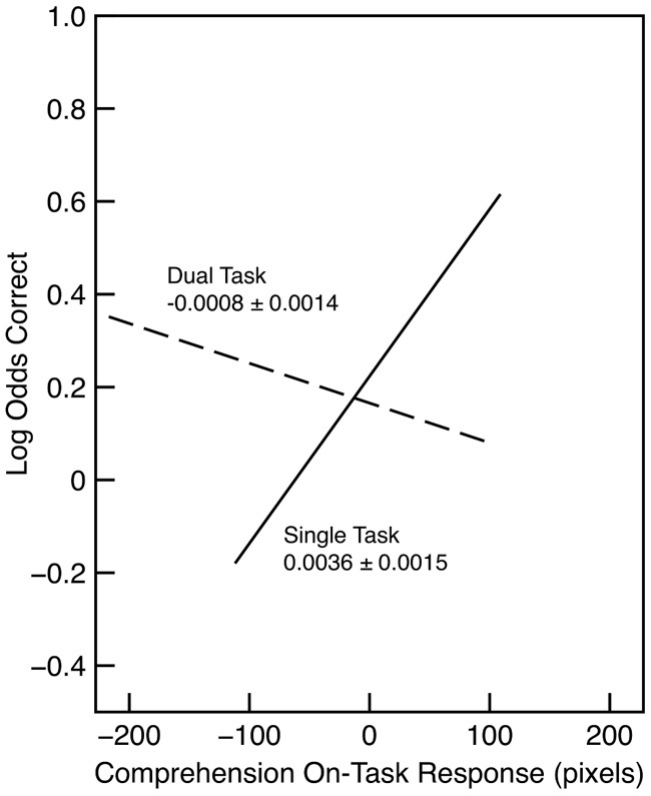
**Comprehension accuracy in Experiment 1 as a function of condition and comprehension on-task probe response**. The slope and standard error is indicated for each regression line. Each line is drawn over the interquartile range of the probe response.

Comprehension accuracy is shown in Figure 1 as a function of the probe response. In this analysis, probe response was treated as a continuous variable, and the figure shows the regression line, over the interquartile range in each condition, as estimated in the analysis. The figure suggests that there is a relationship between comprehension and on-task probe response in the single-task condition but not in the dual-task condition, even though the range of probe responses in the two conditions is comparable. In order to assess the evidence for a difference in relationship between comprehension and on-task probe response, we began with a model of accuracy that included only the effect of condition. We compared that to a model that also included the comprehension on-task probe response as a predictor only in the single-task condition. The latter model was better (λ_adj_ = 5.08), providing evidence for a relationship between comprehension and mind wandering when subjects only have to focus on comprehension. We then compared this model to one that also included probe response as a predictor in the dual-task condition. There was no evidence that adding this predictor improved the model (λ_adj_ = 0.44). There was modest evidence that the effect of on-task probe was different in the two conditions: A model that included the interaction between probe response and condition was better than a model that included only a maineffect of probe response (λ_adj_ = 3.85). In order to provide evidence that there was little effect of the on-task probe in the dual-task condition, we compared a model in which the effect of on-task probe response in the dual task condition was set to 0 to one in which it was constrained to be at least 0.0018 logits/pixel, that is, half the size of the effect in the single-task condition. The latter model provided a clearly better fit (λ_adj_ = 5.10). Finally, for the dual-task condition, there was no evidence that comprehension was related to the probe response for the letter-detection task (λ_adj_ = 0.73; the slope estimated in the model was 0.0020 logits/pixel, *SE* = 0.0015).

As another description of the pattern of results, confidence intervals were generated with parametric bootstrapping. We started with the best-fitting model in which the effect of probe response could differ in the two conditions and then generated 1000 random samples based on that model. The model was then refit to each of these new datasets, and the resulting sample of parameter estimates was used to estimate confidence intervals. Based on this procedure, the 95% confidence interval for the effect of probe response in the dual-task condition was −0.0036 to 0.0019 logits/pixel. This interval is consistent with the model comparison described above that suggested that the effect of probe response in the single-task condition was not substantial. More critically, the 95% confidence interval for the difference between the effect of probe response in the single- and dual-task condition was 0.0035–0.0088 logits/pixel. This interval does not contain zero and is consistent with the previous model comparison that provides evidence for a larger effect of probe response in the single-task condition. On balance, although we cannot discount the possibility that there is a modest effect of the probe response in the dual-task condition, the results indicate at least that such an effect cannot be large. Further, the pattern of results is consistent with an interpretation in which comprehension is a function of on-task response in the single-task condition is larger than that in the dual-task condition.

The relationship between reading comprehension and mind wandering (as measured by on-task probes) has been documented in a number of previous studies, including Schooler et al. (2004); Smallwood et al. (2008), and Dixon and Bortolussi (2013). Thus, the results for the single-task condition provides a convergent replication using a word-by-word reading method. A similar result was reported by Franklin et al. (2011): Subjects who reported more off-task episodes in a word-by-word reading paradigm had lower comprehension scores. This finding is valuable because previous accounts of mind wandering in reading have often assumed that the control of eye movements was relatively automatic and could proceed without active involvement when mind wandering. However, the present requirement of pressing the space bar to advance to the next word would not have the same degree of practice and naturalness. Yet the relationship between comprehension and on-task rating was still observed. Consequently, one must conclude that comprehension can suffer from the lack of resources even when the movement from word to the next is actively controlled.

In contrast to the single-task results, the failure to find strong evidence for this relationship under dual-task conditions is surprising. It seems unlikely that this result occurred because the dual-task condition requires allocating more resources to reading. If this were the case, one might have expected to find generally higher on-task responses in the dual-task condition, but there was little evidence for such a difference. One might also conjecture that the task demands of the letter-detection task required resources to be allocated to word identification, and that this additional resource allocation was sufficient to answer the comprehension on-task probes. However, if this were the case, one might have expected that comprehension would be related to on-task rating for the letter-detection task, and this did not seem to be the case either. As well, there is some reason to suspect that word recognition occurs even during mind wandering (e.g., Reichle et al., 2010). As we argue in the General Discussion, this pattern of results may require a deeper analysis of how subjects respond to on-task probes.

#### Letter detection

The tendency to make false alarms and hits on content words, function words, and the word “the” is shown in Table 3 in terms of the log odds of an “e” response (and the corresponding proportion). Models were fit to the false alarms and the hits separately. For false alarms, we compared a null model to one that included a difference between content and function words. However, the comparison yielded no evidence for an effect of word type (λ_adj_ = 0.69). The data for hits, however, clearly demonstrated the missing-letter effect: hit rate was lower for function words relative to content words and lower still for the word “the.” In particular, a model that included a difference between content and function words was substantially better than the null model in which no difference was predicted (λ_adj_ > 1000), and a model that further included a decrease in hit rate for the word “the” was better still (λ_adj_ = 23.29).

**Table 3:** **“e” Response rate (and Standard Error) as a function of word type in Experiment 1**.

	**Content words**	**Function words (excluding “the”)**	**“the”**
Log odds false alarms	−3.176 (0.050)	−3.288 (0.050)	
Proportion false alarms	0.040 (0.002)	0.036 (0.002)	
Log odds hits	1.348 (0.039)	0.974 (0.048)	0.707 (0.052)
Proportion hits	0.794 (0.006)	0.726 (0.009)	0.670 (0.011)

**Figure 2:**
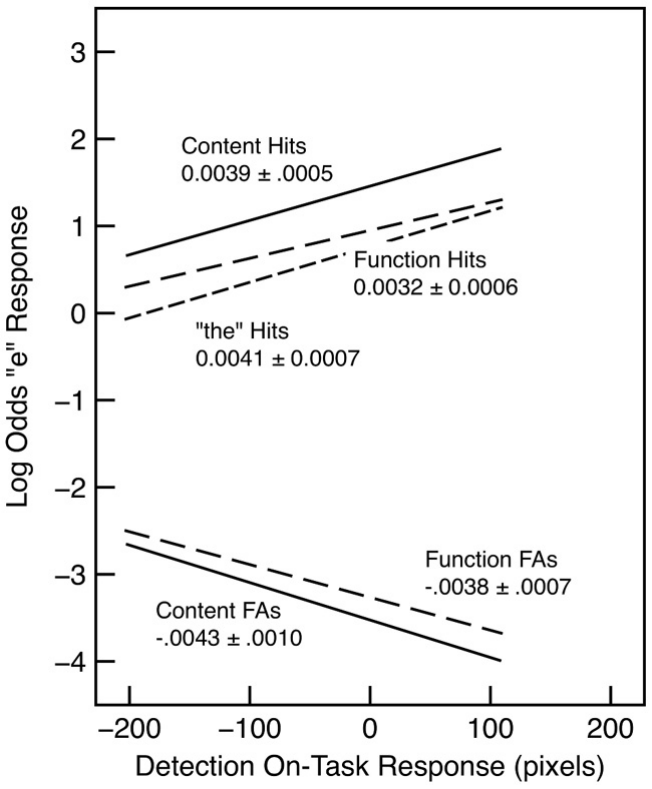
**“e” detection hits and false alarms as a function of word type and detection on-task probe response in Experiment 1**. The slope and standard error is indicated for each regression line. Each line is drawn over the interquartile range of the probe response.

In order to assess the extent to which performance varied with on-task probe response for the letter-detection task, we examined words in the second half of each section delimited by the probes. These results are shown in Figure 2. In general, detection accuracy improved with higher probe responses, as shown by both an increase in hits and a decrease in false alarms. However, these trends did not interact with word type. For both false alarms and hits, a model that included probe response and word type was substantially better than a model that included word type alone (λ_adj_ > 1000 in both cases). There was no evidence that adding the interaction with word type improved the model in either case (λ_adj_ = 0.42 for false alarms; λ_adj_ = 0.31 for hits).

These results replicate the well-known missing-letter effect as found, for example, by Healy (1976) and Greenberg and Koriat (1991). Critically, the results show little evidence for an interaction with on-task rating. In other words, there is just as much of a tendency to miss the letter e in function words when subjects are on task as when they are mind wandering. This means that the missing letter effect cannot be attributed to a failure to devote adequate resources to the orthographic level. An alternative interpretation is that the effect is inherent in the nature of the representations used to perform the task. For example, word recognition processes may be assumed to be largely automatic among skilled readers (Rayner and Pollatsek, 1989), and lexical processing may proceed in the same fashion regardless of how resources are allocated (cf. Brown et al., 2002). However, the representation of the content and function words produced by lexical processing may differ in the extent to which they provide access to the component letters. This is in keeping with the “unitization” account of the effect (Healy, 1994) and with some of the tenets of the GO model. Unlike other aspects of the GO model, the effect observed here cannot be attributed to skipping function words that are recognized in the periphery because all words are presented at fixation (see also Roy-Charland et al., 2009). Similarly, the present results suggest that the missing-letter effect should not be attributed to attention to the letter level as such, since it does not interact with the allocation of resources as indexed by the on-task probes.

Our interpretation is that on-task probe response provides an index of the resources allocated to the decision process required by the e/no-e response made for every word. When resources are devoted to this decision process (i.e., when subjects are on task), detection accuracy increases; more misses occur when fewer resources are devoted to the decision process. An interesting aspect of these results is that a failure to attend to the detection task is associated not only with a decrease in the detection hit rate, but also with an increase in the false alarm rate. This suggests that allocating insufficient resources to the decision process leads to poorer discrimination between the two possible types of stimuli (i.e., words with e's and those without), rather than simply a failure to perform the task.

#### Reading time

Reading time per word is shown for the single- and dual-task conditions as a function of word type in Table 4. Reading was much faster in the single-task condition, and a model that incorporated this effect was much better than the null model (λ_adj_ > 1000). Oliver et al. (2005) found comparable results on reading time even without the present word-by-word reading time constraints. In the slower dual-task condition, content words were read more slowly than function words. Adding this effect improved the model substantially (λ_adj_ > 1000). However, there was little evidence for an effect of word type in the single-task condition; that is, adding an effect of word type in the single task condition did not improve the model appreciably (λ_adj_ = 1.41).

**Table 4:** **Reading time per word in seconds (and Standard Error) as a function of condition and word type in Experiment 1**.

**Word type**	**Single-task condition**	**Dual-task condition**
Content	0.389 (0.027)	0.728 (0.027)
Function	0.376 (0.027)	0.627 (0.027)

**Figure 3:**
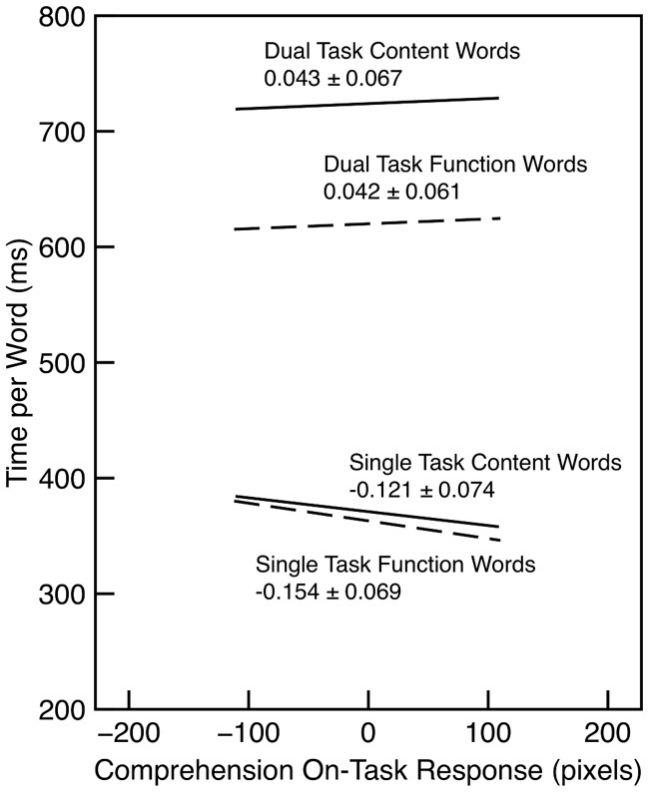
**Reading time per word as a function of word type and comprehension on-task probe response in Experiment 1**. The slope and standard error is indicated for each regression line. Each line is drawn over the interquartile range of the probe response.

The relationship between reading time and on-task probe response was examined for words in the last half of each section. In the single task condition, there was tendency for reading time to decrease as on-task rating increased (λ_adj_ = 5.30). A similar effect was observed by Franklin et al. (2011) using a word-by-word reading paradigm. (However, Reichle et al. (2010) found the opposite result—slower reading while mind wandering—under more naturalistic reading conditions.) There was no such trend for the dual-task condition (λ_adj_ = 0.52). These trends are illustrated in Figure 3.

The reading times illuminate some aspects of how readers coordinate the task of reading comprehension with the task of detecting letters. As discussed previously, the on-task probes for comprehension and letter detection do not provide a strong indication of a tradeoff between the two tasks in the dual-task condition; in fact, there was a weak, positive relationship. However, performing both tasks together clearly involves more work, and the reading times reflect that: Reading time was much faster in the single-task condition. We hypothesize that there is an additional decision and response selection process that is added for each word in the dual-task condition, and these processes consume an additional several hundred milliseconds.

Although reading times in the dual-task condition were shorter for function words than for content words, both types of words were substantially slower than reading times in the single-task condition. Thus, it would be difficult to argue that there was insufficient time to identify the letters in the word under dual-task conditions. This is consistent with our previous argument that the missing-letter effect derives from the nature of the representation of the recognized words, not the resources or time devoted to making a letter-detection decision about that representation.

## Experiment 2

The surprising failure to find a relationship between mind wandering and comprehension under dual-task conditions in Experiment 1 may be related to the nature of the comprehension measure. Indeed, as mentioned previously, recognition memory might be based on the results of meaning processing, and it is often assumed that mind wandering interferes with the construction of a situation model, not meaning processing (e.g., Smallwood et al., 2008). In particular, we conjecture that the comprehension items used in Experiment 1 tapped a relatively superficial representation of the text, and, in many cases, the items could have been answered correctly if subjects recognized a word or phrase. Thus, a situation model often would not be needed for a correct response. If this analysis is correct, the relationship between on-task rating and comprehension should re-emerge using a measure of comprehension that taps the reader's situation model rather than recognition of semantic content. This might be done, for example, by carefully designing multiple-choice questions to interrogate inferences and relationships that were not explicitly stated and would only be available in the situation model. However, Dixon and Bortolussi (2013) argued that measuring passage recall was more likely to be based on the situation model representation than recognition memory because it requires an organized and articulated representation of the story world events (see also Ericsson and Kintsch, 1995). This hypothesis was tested in Experiment 2.

### Method

#### Procedure

The presentation of the text was precisely the same as in Experiment 1, and, for the dual-task subjects, the letter-detection task was performed in the same manner. After reading the text, subjects were provided with a computer document in the Apple TextEdit program with the instruction to type a summary of the story, including as much detail as possible. There was no limit on the time taken to produce the summary. Subjects were informed at the outset that they would have to write a summary of the passage.

#### Subjects

Subjects were paid volunteers recruited from undergraduate Psychology classes at the University of Alberta. There were 19 subjects in the both the dual- and the single-task conditions. However, data from one dual-task subject was omitted because of a false alarm rate of over 80% on the letter-detection task. The treatment of subjects, including procedures for obtaining informed consent, was approved by the Arts, Science, and Law Research Ethics Board at the University of Alberta according to the provisions of the Canadian Tri-Council Policy Statement, “Ethical Conduct for Research Involving Humans.”

#### Analysis

Reading time and e detection were analyzed as in Experiment 1. Recall performance was scored by first dividing each recall protocol into statements. Each statement generally consisted of a single clause from the protocol but omitting those that had little to do with the content of the text (e.g., “I think”). Verb forms that did not entail a separate clause but which nevertheless referred to a distinct event in the story were also considered statements (e.g., “being knighted by Prince Charles.”) A liberal criterion was adopted for scoring the recall statements: A statement was omitted only if the information was completely incorrect or if it was so general that it could be attributed to any section in the text (e.g., “The excerpt from the novel talked quite favorably of this Kenelem Digby gentlemen”). We then counted, for each half section of the text, the number of statements that could have been based on the information from that portion of the text. (Because some statements could have been based on the material from several different places in the text, the total of these counts was larger than the number of statements.) The parsing into statements and the attributions to section was done independently by two research assistants and discrepancies were resolved in consultation with the first author. The count data were analyzed using generalized linear models using the Poisson family and a log link function. (This analytical technique is often recommended for count data; e.g., Zuur et al., 2009). Thus, the comprehension dependent variable and associated model parameters are expressed in terms of log counts.

### Results and discussion

#### On-task probes

The mean response for comprehension on-task probe (in pixels) was 35.6 (*SE* = 17.0 across subjects) in the dual-task condition and 34.4 (*SE* = 27.35 across subjects) in the single-task condition. The standard deviation of responses within subjects was 96.0. The mean response for the letter-detection on-task probe was 83.7 (*SE* = 15.6 across subjects), and the standard deviation within subjects was 80.3. There was clear evidence for a positive relationship between the letter-detection responses and the comprehension responses (λ_adj_ = 8.94). After controlling for the mean response level for each subject, the correlation between the letter-detection response and the comprehension response was 0.372.

#### Recall

The number of recall statements based on each section is shown in Table 5, both as a log count (derived from the fit of the generalized linear model) and the corresponding raw counts. There was no evidence for higher recall in the single-task condition (λ_adj_ = 0.70).

**Table 5:** **Recall statements per section (and Standard Error) in Experiment 2**.

	**Single-task condition**	**Dual-task condition**
Log count	1.269 (0.061)	1.129 (0.061)
Count	3.56 (0.22)	3.09 (0.19)

**Figure 4:**
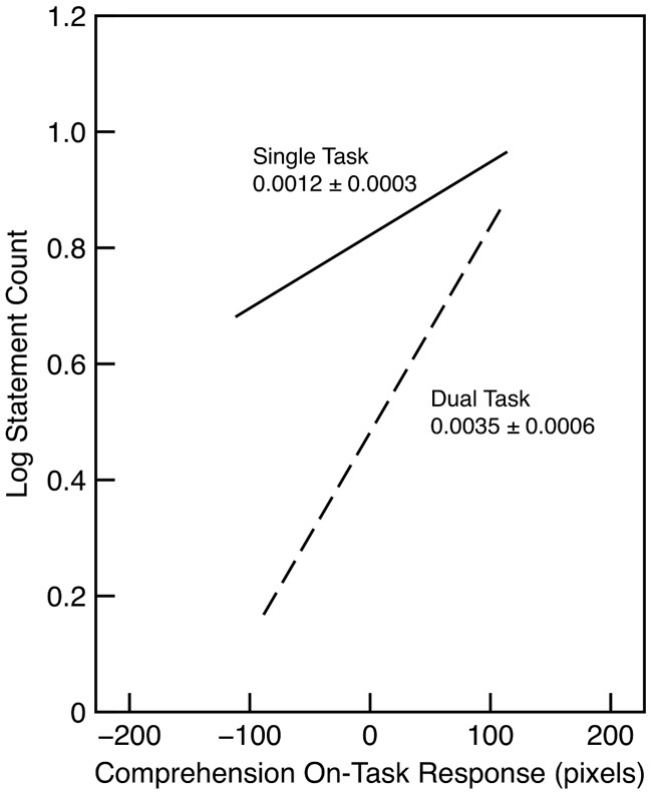
**Comprehension accuracy in Experiment 2 as a function of condition and comprehension on-task probe response**. The slope and standard error is indicated for each regression line. Each line is drawn over the interquartile range of the probe response.

The relationship between recall and comprehension on-task probe was examined by looking at the number of recall statements based on material in the last half of each section. Figure 4 shows the results of this analysis, treating probe response as a continuous variable. The figure shows the regression lines in each condition, over the respective interquartile range, as estimated in the generalized linear mixed-effects analysis. A model in which the probe response predicted accuracy in the single-task condition was better than a model that only included the effect of condition (λ_adj_ = 27.30). There was also strong evidence for an effect of the probe response in the dual-task condition (λ_adj_ > 1000). Indeed, there was good evidence that the effect in the dual-task condition was actually larger than that in the single-task condition (λ_adj_ = 110.89). In the dual-task condition, there was no evidence that comprehension was related to the probe response for the letter-detection task (λ_adj_ = 1.04). Thus, the results show that comprehension improved with on-task rating in both the single-task and the dual-task conditions.

The results replicate previous findings that recall is better when subjects are on task. For example, Dixon and Bortolussi (2013) found a similar relationship between on-task probe response and recall. Unlike the results for recognition memory in Experiment 1, however, this result was even stronger in the dual-task condition than in the single-task condition. Thus, it seems clear that the unexpected result in Experiment 1 was due to the nature of the memory measure. Of course, this does not explain why the relationship with on-task probe should interact with single-/dual-task; this issue is taken up in the General Discussion.

A potential concern with this interpretation is that the failure to find a relationship between probe and recognition accuracy in Experiment 1 may have been due to the smaller sample and hence lower power of that experiment. In order to address this issue, we analyzed the memory results from the dual-task condition of both experiments together. However, a problem in performing such an analysis is that the dependent measures in the two experiments are different: Each probe response in Experiment 1 was associated with two multiple choice items (each of which could be correct or incorrect), while each probe response in Experiment 2 was associated with a recall statement count. To put the two dependent variables on the same scale, we coded memory performance for each probe in both experiments as either high or low. High performance in Experiment 1 was defined as getting both multiple choice items correct; high performance in Experiment 2 was defined as recalling more than 2 statements from the material prior to the probe. Although there is no reason to think that the overall level of high performance using these criteria should be similar in the two experiments, the index should allow a meaningful comparison of the effect of probe response across experiments.

Analysis of high vs. low comprehension in the dual-task conditions was done by fitting generalized mixed-effects models using the binomial family and a logistic link function. A model that included an overall effect of probe response was better than a model that included merely the effect of experiment (λ_adj_ = 7.09). However, a model that included an interaction with experiment was substantially better (λ_adj_ > 1000). In particular, adding an effect of probe for Experiment 2 alone lead to a substantial improvement in the model (λ_adj_ > 1000), but there was no further improvement when the probe response for Experiment 1 was added (λ_adj_ = 0.68). In contrast, a similar analysis of the single-task conditions in the two experiments provided evidence for an overall effect of probe (λ_adj_ = 7.68) but no evidence for an interaction with experiment (λ_adj_ = 0.48). Thus, there is good evidence that in the dual-task conditions, the effect of probe on memory was larger in Experiment 2 (using a recall measure) than in Experiment 1 (using a recognition measure). The differential results in the two experiments cannot be attributed to a difference in power.

#### Letter detection

The tendencies to make false alarms and hits on content words, function words, and the word “the” are shown in Table 6 in terms of log odds of an e response (as well as the corresponding proportions). Models were fit to the false alarms and the hits separately. For false alarms, there was no evidence for a difference between content and function words (λ_adj_ = 0.38). The data for hits, however, clearly demonstrated the missing-letter effect, with a lower hit rate for function words relative to content words. A model that included a difference between content and function words was substantially better than the null model in which no difference was predicted (λ_adj_ > 1000). However, there was little evidence for a further decrease in hits for the word “the” (λ_adj_ = 1.28).

**Table 6:** **“e” Response rate (and Standard Error) as a function of word type in Experiment 2**.

	**Content words**	**Function words (excluding “the”)**	**“the”**
Log odds false alarms	−3.852 (0.049)	−3.882 (0.049)	
Proportion false alarms	0.021 (0.001)	0.020 (0.001)	
Log odds hits	2.487 (0.034)	1.716 (0.039)	1.598 (0.042)
Proportion hits	0.923 (0.002)	0.848 (0.005)	0.831 (0.006)

**Figure 5:**
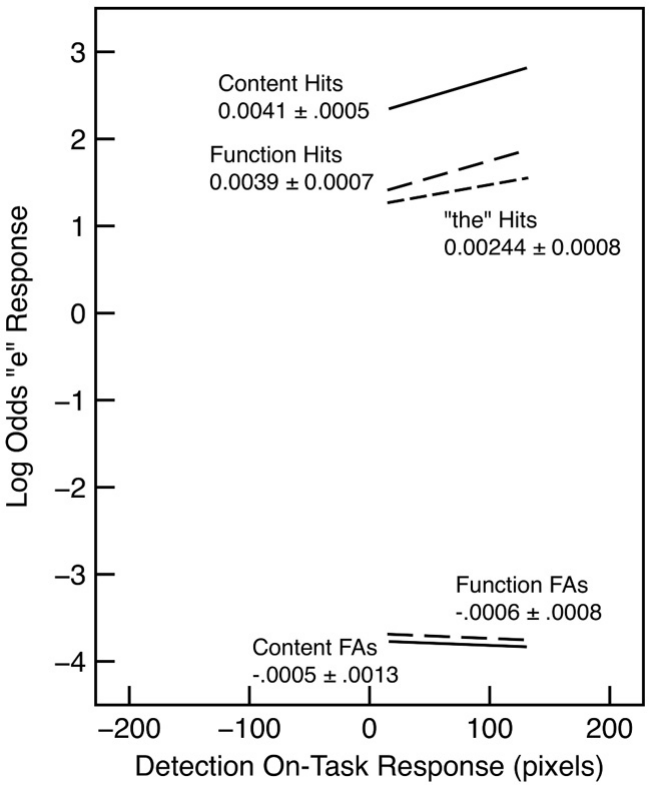
**“e” detection hits and false alarms as a function of word type and detection on-task probe response in Experiment 2**. The slope and standard error is indicated for each regression line. Each line is drawn over the interquartile range of the probe response.

In order to assess the extent to which letter detection varied with letter-detection on-task probe response, we examined words in the second half of each section delimited by the probes. These results are shown in Figure 5. In general, the hit rate improved with higher probe responses, although there was little effect on false alarms. For hit rates, a model that included word type was substantially better than a model that only included word type (λ_adj_ > 1000). There was no evidence that the effect of probe interacted with word type (λ_adj_ = 1.01). For false alarms, there was no evidence of an effect of on-task probe (λ_adj_ = 0.47) and no evidence for an interaction between probe response and word type (λ_adj_ = 0.37).

The results for the letter-detection task in Experiment 2 differ somewhat from those in Experiment 1. Generally, subjects reported being on task to a greater degree, and there was a much more restricted range for the probe responses. Overall, performance was better. Subjects in this experiment were recruited differently at a different time of the academic year, and we suspect that they generally had a higher degree of motivation than the subjects in Experiment 1. This difference in sampling may have been responsible for the higher level of performance. Nevertheless, the overall pattern was the same: Detection performance increased with on-task rating, but the missing-letter effect was obtained regardless of whether or not subjects indicated they were on task. Although an effect of on-task rating was found for false alarms in Experiment 1, no such relationship was found here. This may be ascribed to both the very low level of false alarms as well as the restricted range of on-task ratings.

#### Reading time

Reading time per word is shown for the single- and dual-task conditions as a function of word type in Table 7. Reading was much faster in the single-task condition, and a model that incorporated this effect was much better than the null model (λ_adj_ > 1000). Content words were read more slowly than function words in both conditions (λ_adj_ > 1000), but this effect was much larger in the dual-task condition (λ_adj_ > 1000).

**Table 7:** **Reading time per word in seconds (and Standard Error) as a function of condition and word type in Experiment 2**.

**Word type**	**Single-task condition**	**Dual-task condition**
Content	0.509 (0.021)	0.767 (0.021)
Function	0.454 (0.021)	0.642 (0.021)

**Figure 6:**
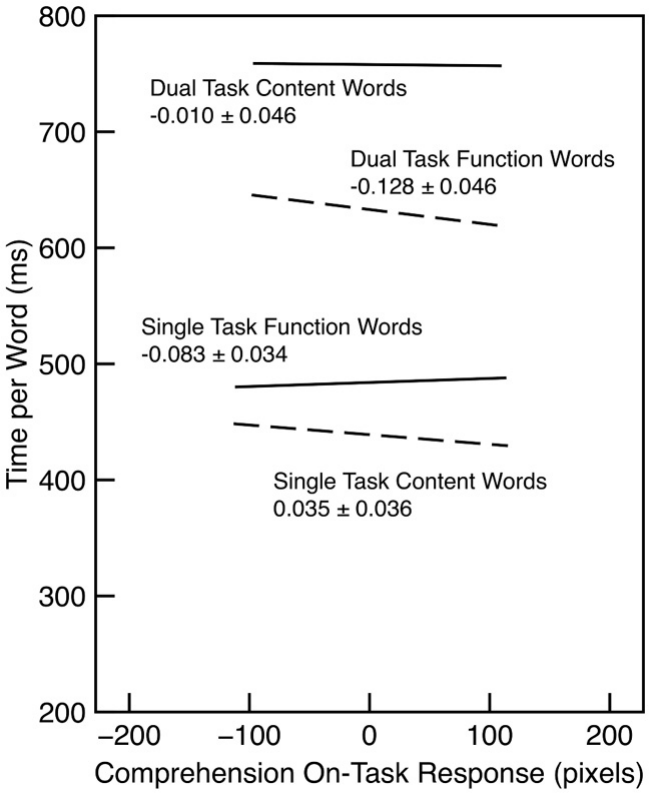
**Reading time per word as a function of word type and comprehension on-task probe response in Experiment 2**. The slope and standard error is indicated for each regression line. Each line is drawn over the interquartile range of the probe response.

The relationship between reading time and on-task probe response was examined for words in the last half of each section. In both conditions, reading time for function words decreased as on-task rating increased (λ_adj_ > 1000). However, there was no evidence for any change in reading time for content words (λ_adj_ = 0.44). These trends are illustrated in Figure 6.

The reading time results are broadly consistent with those from Experiment 1: Reading times were substantially slower in the dual-task condition, and the difference between content and function words was substantially smaller in the single-task condition. As argued previously, subjects in this experiment seemed to be more motivated than those in Experiment 1, and possibly they devoted more effort to performing the task. This may have lead to the slower reading times in the single-task condition and the larger effect of word type in that condition. The pattern of reading times is consistent with the idea that readers process words less fully when they are off task (cf. Reichle et al., 2010; Schad et al., 2012). In the present context, this could have produced a smaller difference between function and content words.

## General discussion

The results of these experiments bear on three issues: the interpretation of on-task probes, the allocation of resources in reading comprehension, and the nature of the missing-letter effect in letter detection. Each will be discussed in turn.

### On-task probes

Our interpretation of the present results rests on the assumption that subjects do not have direct access to how resources have been recently allocated. Instead, we argue that resource allocation is a largely unconscious mechanism that responds to the constantly changing demands of the situation and current processing. Although deliberate intentions certainly matter in resource allocation, they do not completely determine allocation; other factors are important as well and may alter resource allocation on a moment-to-moment basis. Indeed, the phenomenon of “zoning out” while reading, even though the primary goal is to understand the text, indicates that resource allocation is not inevitably tied to deliberate goals. If, as we argue, resource allocation is generally unconscious and unavailable to introspection, responding to on-task probes requires some form of inference. Our hypothesis is that readers solve this inferential problem in reading by assessing the contents of working memory. In particular, if they find that working memory contains detailed and elaborate information about the situation described by the text, they may infer that they must have been on task. On the other hand, if the representation text is relatively impoverished, they may conclude that they were mind wandering. This interpretation ties the on-task response to well-understood processes in working memory without assuming that readers have a special insight into their mental state at any given point in time. Moreover, this analysis provides an account of the pattern of results across the two experiments and two reading conditions, as discussed below.

### Resources in reading

A complex task such as reading requires the coordination of resource allocation to a range of different processes. As outlined in the introduction, this idea has been articulated by LaBerge and Samuels (1974), Just and Carpenter (1992), and Dixon and Bortolussi (2013), among others. In the interpretation of the present results, it is important to distinguish among resources allocated to processes that construct a meaning representation from those that construct a situation model. Further, we assume that the memory measures in Experiment 1 and 2 are differentially affected by the processing of meaning and by the processing of the situation model. In particular, recall seems likely to depend heavily on a well-developed situation model (e.g., Ericsson and Kintsch, 1995). Together with the argument made previously that on-task probes index the richness of the situation model representation in working memory, this analysis easily explains the recall results in Experiment 2: Subjects report being on task when they have an elaborate situation model, and that situation model allows them to recall information from the text.

According to this analysis, it is not surprising that recognition performance in the dual-task condition of Experiment 1 failed to show a relationship with on-task probe response. Because the recognition task required only a superficial understanding of the material and many items could be answered by recognizing a particular concept or event, there is no reason to expect the representation of the situation model to predict comprehension performance. This argument does not mean that recognition would always be accurate—for many recognition items, the relevant concept or event may be insufficiently active to distinguish it from the foils. Rather, the claim is that this activation is not directly indexed by the quality of the situation model representation. From this perspective, the aspect of Experiment 1 that is more surprising is that there *was* such a relationship in the single-task condition. Our interpretation, though, is that under these conditions, there is a correlation between the allocation of resources to meaning representations and allocation to the situation model. Generally, when a reader is off task, fewer resources would be allocated to both; when a reader is on task, more resources would be allocated to both. Thus, the nature of the situation model would provide a reasonable index of subsequent recognition memory, despite the fact that the on-task probes are not indexing precisely the mental content that is relevant for subsequent memory. In contrast, in the dual-task condition, when resources are not allocated to the situation model, they might instead be allocated to the processes involved in letter detection. In particular, word meanings may be activated in the course of performing the letter detection task, and the activation of these concepts could provide a basis for performing the subsequent recognition test, even if the situation model representation is relatively impoverished.

Dixon and Bortolussi (2013) provided converging evidence for this analysis by probing not just whether or not readers were on task, but also whether or not they were deeply involved with the story world. Again, this response would have to be made by interrogating the contents of working memory. However, this “involvement” probe question is arguably more germane to the nature of the situation model in working memory and might provide a better index of the extent and detail of this representation. In keeping with this assumption, Dixon and Bortolussi found that the involvement probe was more closely related to subsequent recall than the on-task probe. In other words, both on-task and involvement responses required inferences, but the inferences required to make involvement responses provided a better index of the information used in recall.

The idea that on-task probe responses are inferences has implications beyond the current pattern of results. One such implication is that if some other variable affects the content of working memory, it may affect the responses readers make to on-task probes even if there is no effect on resource allocation. For example, if the reader has extensive background knowledge relevant to a text, the situation model is likely to be more detailed and elaborate, and he or she may be less likely to report being off task given the same pattern of resource allocation. Interest value of the text may operate similarly. Although it is certainly plausible to assume that more interesting texts will lead to lower rates of mind wandering, it is possible that more interesting texts lead to more detailed situation model representations for other reasons and consequently a higher rate of reporting being on task. In tasks other than reading comprehension, subjects may make inferences about being on task using different sources of information. For example, in the SART task, subjects may decide that they were mind wandering if they can recall missing targets. Consequently, any manipulation that makes the errors more memorable should increase the rate of reported mind wandering. Another example is listening to lectures (Risko et al., 2011); in this case, subjects may make on-task inferences by assessing whether they have a coherent representation of the lecture content. If subjects adopted such a strategy, more organized material might lead to lower rates of reported mind wandering. The overarching conclusion is that one cannot assume that there is a simple relationship between on-task ratings and the allocation of resources.

### The missing-letter task

To our knowledge, the on-task probe technique has not been previously combined with the letter-detection task. The most important finding for accounts of the missing-letter effect is that mind wandering impairs letter detection but does not affect the magnitude of the effect. This result is consistent with analyses of the task that depend on the nature of the activated word representation. Consistent with the unitization mechanism proposed by Healy (1994) and as incorporated in the GO model (Greenberg et al., 2004), we assume that the word representations generated in reading may not contain detailed information about constituent letters, particularly if the words are high-frequency function words. In the present experiments, devoting resources to the letter-detection task does not change this; it merely means that the readers will make better decisions concerning that representation. This produces a higher hit rate and (in Experiment 1) a lower false alarm rate.

An important control condition for the missing-letter effect is to scan lists of unrelated words for a particular letter. Healy (1976) found that when subjects read scrambled text, a missing-letter effect was still obtained, consistent with the argument that the role of function words in well-formed sentences was not essential to observe the effect. This is consistent with the unitization mechanism and our interpretation of the present data. Broadly speaking, we argue that the orthography of function words is less salient in the initial representation produced by lexical processes. Following from this logic, we predict that the pattern of results obtained here would also be observed with scrambled text or in other conditions in which readers need not process the content of sentences. In particular, we would expect to see a relationship between letter-detection accuracy and the response to on-task probes but no interaction with the missing-letter effect. As here, the effect should be observed regardless of whether subjects report being on task or not.

An innovation in the current investigation of the missing-letter effect is the use of generalized linear models to examine the interactions with on-task probe. Similar to logistic regression techniques, this approach is immune to the types of scaling artifacts that arise in the analysis of proportion correct (Everitt, 2001; Dixon, 2008). In some previous research on the missing-letter effect, an interaction with the magnitude of the effect was obtained largely because the manipulation affected the overall level of performance, and the magnitude of the missing-letter effect may have been influenced by such scaling effects (e.g., Saint-Aubin et al., 2010).

## Conclusions

Because mind wandering involves the allocation of resources to task-unrelated processes, it is useful to analyze performance under conditions that require subjects to perform more than a single task. The present results inform our understanding of the on-task probe technique, the allocation of resources to the multiple components of reading comprehension, and the nature of the representations used in the letter-detection task.

### Conflict of interest statement

The authors declare that the research was conducted in the absence of any commercial or financial relationships that could be construed as a potential conflict of interest.
